# Volatility-driven learning in human infants

**DOI:** 10.1126/sciadv.adu2014

**Published:** 2025-06-25

**Authors:** Francesco Poli, Tommaso Ghilardi, Jana H. M. Bersee, Rogier B. Mars, Sabine Hunnius

**Affiliations:** ^1^ MRC Cognition and Brain Sciences Unit, University of Cambridge, Cambridge, UK.; ^2^ Donders Institute for Brain, Cognition and Behaviour, Radboud University, Nijmegen, Netherlands.; ^3^ Centre for Brain and Cognitive Development, Birkbeck, University of London, London, UK.; ^4^ University of Amsterdam, Amsterdam, Netherlands.; ^5^ Wellcome Centre for Integrative Neuroimaging, Centre for Functional MRI of the Brain (FMRIB), Nuffield Department of Clinical Neurosciences, John Radcliffe Hospital, University of Oxford, Oxford, UK.

## Abstract

Adapting to change is a fundamental feature of human learning, yet its developmental origins remain elusive. We developed an experimental and computational approach to track infants’ adaptive learning processes via pupil size, an indicator of tonic and phasic noradrenergic activity. We found that 8-month-old infants’ tonic pupil size mirrored trial-by-trial fluctuations in environmental volatility, while phasic pupil responses revealed that infants used this information to dynamically optimize their learning. This adaptive strategy resulted in successful task performance, as evidenced by anticipatory looking toward correct target locations. The ability to estimate volatility varied significantly across infants, and these individual differences were related to infant temperament, indicating early links between cognitive adaptation and emotional responsivity. These findings demonstrate that infants actively adapt to environmental change, and that early differences in this capacity may have profound implications for long-term cognitive and psychosocial development.

## Teaser

Infants estimate environmental volatility to learn optimally, with early individual differences linked to temperament.

## INTRODUCTION

The capacity to flexibly adapt to change is essential for enduring, growing, and thriving in the face of life’s challenges. In the last 20 years, research has identified the cognitive and neural mechanisms underlying this adaptive learning process (*1*–*3*). When humans encounter unexpected outcomes, how they respond depends critically on whether the environment is stable or volatile. In stable environments, unexpected outcomes can be treated as noise, leading learners to maintain or “exploit” previously acquired beliefs. In contrast, in volatile environments, unexpected outcomes are more likely to signal genuine changes in contingencies, prompting learners to adjust or replace existing beliefs with new ones. In the context of this paper, volatility refers to an experimental manipulation that creates unstable or changing conditions, thereby allowing us to investigate humans’ ability to adapt to change (*4*).

Although studied extensively in adults, the developmental roots of this ability are still unknown. Current research assumes that unstable environments impact psychological development (*5*–*7*). In volatile environments, children display higher vigilance (*8*), are more exploitative (*9*), act more impulsively (*10*), and favor instant over delayed rewards (*11*). Recent work shows that volatile environments do not only affect immediate behavior, but also affect brain development, changing the brain’s structure and connectivity (*12*, *13*) with a cascade of potential consequences for later cognitive development and psychosocial well-being (*14*–*16*). Tracing the ability to adapt to change back to infancy could substantially shift our perspective on the active (rather than passive) nature of infant development, highlighting how infants actively tackle unexpected changes in the environment, rather than simply focusing on how they react to and are affected by such changes.

Identifying individual differences in how infants adapt to change can advance our understanding of developmental trajectories for psychological well-being. Volatility estimation shapes how we perceive and respond to changes in our daily lives, influencing our capacity to adapt to new circumstances. In adults, individual differences in volatility estimation have been linked to multiple mental health conditions, to the extent that volatility misestimation has been proposed as a transdiagnostic process that broadly affects mental health (*3*). Consequently, a closer look at individual differences in infants’ volatility estimation could shed light on the early mechanisms underlying cognitive resilience and vulnerability (*17*). To investigate the link between infants’ volatility estimation and early indicators of psychosocial well-being, we collected parental reports of infant temperament. Difficult temperament is a stable trait throughout infancy (*18*, *19*), and it has long been viewed as a risk factor for poorer psychosocial outcomes later in life (*20*, *21*), making it a useful proxy measure of well-being in infancy.

**Fig. 1.**
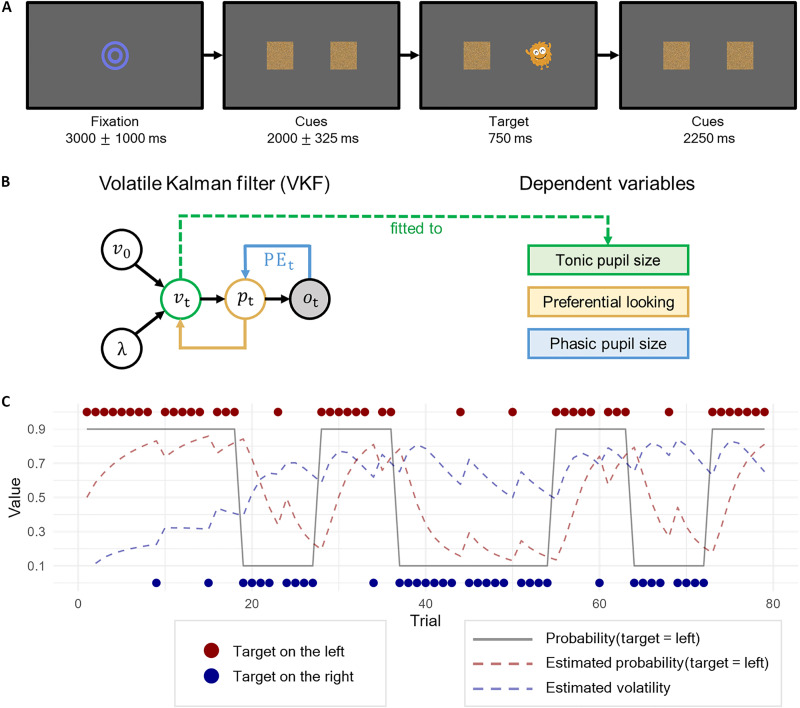
A model-based approach to volatility estimation in infants. (**A**) The reversal learning task involves multiple trials where a target stimulus appears from one of two locations. The predictability of the most likely target location was manipulated, such that it could either be stable (e.g., first 18 trials) or change more frequently (e.g., in trials between 19 and 36) thereby introducing volatility. (**B**) A schematic representation of the computational model and the dependent measures collected from infants’ gaze and pupil data. The model was a volatile Kalman filter (VKF), where $v_{0}$ indicates the initial volatility, λ is the volatility learning rate, $v_{t}$ is the volatility of each trial, $p_{t}$ is the belief about the target location, and $o_{t}$ is the observed outcome of the target location. Observing the outcome would change the internal belief about the target location, as well as higher order beliefs about how volatile the environment is. In turn, these higher-order beliefs would guide future predictions about the target location. (**C**) The target locations order (red and blue dots) with the VKF’s probability estimates about the target location (red line) and the VKF’s volatility estimates (blue line) from an example sequence.

In this study, we used an experimental paradigm, pupillometric data, and a computational modeling approach (Fig. 1) to identify the early origins of volatility estimation in humans, shedding light on the active role played by infants in responding to environmental change. We show that infants are capable of actively adjusting their learning in response to volatility, rather than being at the mercy of (and reactive to) external events. In addition, we detected individual differences in infants’ volatility estimation, demonstrating that variability in this ability is present from early on, and differences therein might thus affect psychosocial development from infancy. In support of this, we show a relationship between infants’ volatility estimation abilities and their temperament.

## RESULTS

### Modeling approach

Eight-month-old infants were presented with a reversal learning task while their pupil size and gaze data were collected (Fig. 1A). To capture their trial-by-trial learning, we used a volatile Kalman filter (VKF) (*22*) (Fig. 1B). The VKF maintains a belief $p_{t}$ representing the probability that the target will appear on a specific location (e.g., the right side). After each trial, the model computes a prediction error ${\text{PE}}_{t}$ , which is the difference between the observed outcome $o_{t}$ and the prior belief $p_{t}$ . This prediction error updates the belief for the subsequent trial, thereby refining the model’s estimates of where the target is likely to appear. In addition, the VKF keeps track of environmental volatility $v_{t}$ which reflects how prone the underlying contingencies are to changing. This volatility estimate is updated based on the model’s recent changes in beliefs $p_{t}$ and is governed by the learning rate parameter λ . Crucially, $v_{t}$ then determines how strongly to weight new information: When volatility is high, large deviations are more likely to be genuine signals of change and thus produce faster updates of the belief $p_{t}$ , whereas under stable conditions, such deviations are more often treated as noise, leading to more conservative updates. A more detailed specification of the model is available in Materials and Methods.

The levels of environmental volatility ( $v_{t}$ ) and the size of the prediction errors ( ${\text{PE}}_{t}$ ) experienced by the infants can be measured via tonic and phasic pupil dilation, respectively. Specifically, tonic pupil size (baseline pupil values persisting over longer timescales) has been associated with heightened subjective uncertainty (*23*, *24*), and volatility can be understood as a second-order form of uncertainty (*3*). In contrast, phasic or transient pupil responses to task-relevant unexpected events are closely linked to prediction-error processing (*23*) and the subsequent updating of internal beliefs (*25*, *26*). These pupillometric signals map onto the tonic and phasic firing modes of the locus coeruleus (*27*–*29*), which are thought to regulate sustained arousal and vigilance (tonic mode) and rapid, event-driven shifts in attention or learning (phasic mode) (*30*).

While optimal learner models are typically fitted to behavioral data (e.g., decision responses), we fitted the VKF directly to a physiological measure using a generalized additive process (Fig. 1B). Specifically, tonic pupil size at each trial’s initial fixation period was used to estimate the model’s initial volatility ( $v_{0}$ ) and learning rate ( λ ) (see Materials and Methods). In addition, baseline-corrected phasic pupil dilation during and after target presentation was taken as a measure of prediction error. Last, preferential looking was computed as the proportion of looking to the cue locations (left cue versus both cues) in the time window starting from the appearance of the two cues to the moment the target appeared. Preferential looking was used to assess the infants’ belief about where the target will appear (*31*).

### Infants track environmental volatility

**Fig. 2.**
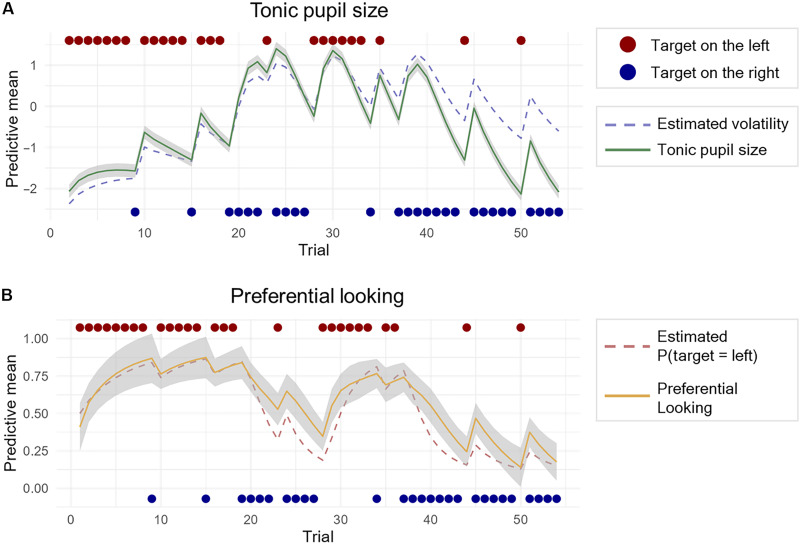
Infants’ tonic pupil size and preferential looking. (**A**) Infants’ tonic pupil size correlated with the VKF model’s estimates of volatility, indicating that they successfully tracked fluctuations in environmental volatility across the task. (**B**) Infants’ preferential looking before target appearance correlated with the VKF model’s predictions about the target location in both stable and volatile periods. This shows that infants were able to integrate volatility information to update their expectations about the target location. Predictive means (*y* axes) were obtained generating trial-by-trial estimates of pupil size and proportion of anticipatory looking from the β coefficients of the fitted regression models.

The model estimates of volatility significantly correlated with infants’ tonic pupil size (*t* = 139.17, β = 0.23, SE = 0.001, and *P* < 0.001), indicating that infants were successfully tracking environmental volatility (Fig. 2A). Specifically, the parameter estimates that best predicted infants’ tonic pupil size were λ (learning rate) = 0.30 and $v_{0}$ (initial volatility) = 0.01. These values indicate that infants started the task expecting a stable environment (i.e., low initial volatility), and successfully adapted their beliefs about environmental volatility as the environment changed, as indicated by levels of λ different from zero.

The model predictions about the most likely target location significantly correlated with infants’ anticipatory looking (*t* = 4.02, β = 1.63, SE = 0.40, and *P* < 0.001) (Fig. 2B). This indicates that, by tracking environmental volatility, infants flexibly adjusted their predictions about where the target was most likely to appear. Crucially, infants successfully predicted the target locations not only in stable but also in volatile environments. This demonstrates that infants were not simply more uncertain or confused when volatility was high, but were instrumentally using the information about environmental volatility to optimize their learning. To strengthen our results, we replicated all the analyses carried out with the initial sample of infants (*n* = 61) with an additional sample of infants (*n* = 30). All effects were successfully replicated (see Materials and Methods).

### Infants optimize their learning

To shed light on the mechanisms that underlie the optimization of learning in changing environments, we analyzed the relation between infants’ phasic pupil size and the trial-by-trial prediction errors estimated by the VKF model. In volatile environments, high prediction errors likely signal a change in the observed regularities and should thus have a large impact on the observer’s existing expectations. Conversely, in stable environments, high prediction errors are likely to be isolated instances that should be disregarded.

**Fig. 3.**
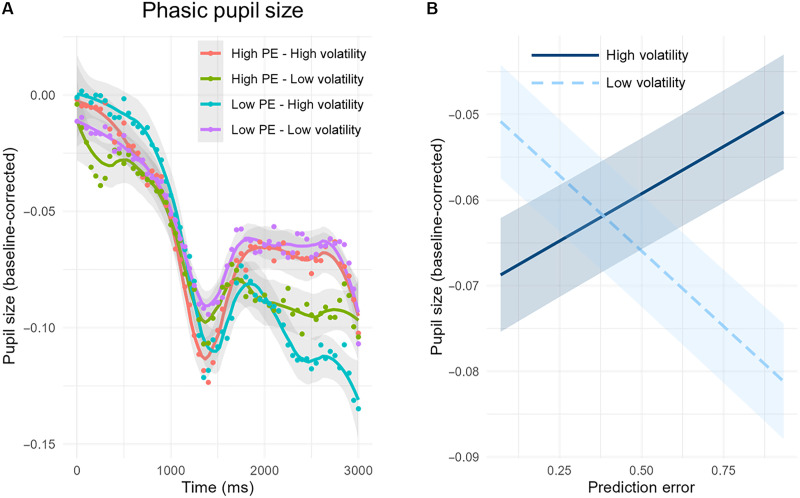
Infants’ phasic pupil size. (**A**) Raw data for phasic pupil size (baseline corrected) at the moment of the target presentation, divided in four groups depending on volatility (mean split in high or low) and the magnitude of the prediction error (big or small). The initial, apparent decrease in pupil size is due to saccades to the target locations on the lateral portions of the screen (0 to 1400 ms). Following this, the slower task-evoked pupil responses emerge (after 1400 ms). (**B**) Predictive means of phasic pupil size as a function of environmental volatility and the magnitude of the prediction errors. Infants considered volatility information when estimating the relevance of prediction errors, such that greater prediction errors were considered as more important when volatility was high (as they might signal a change in the environment). Conversely, smaller prediction errors were considered as more important when the environment was stable (as they corroborated existing beliefs about the target location).

Consistent with these predictions, the interaction between volatility and the magnitude of the prediction errors significantly modulated phasic pupil size (*t* = 14.71, β = 0.10, SE = 0.006, and *P* < 0.001). As depicted in Fig. 3, when the environment was more volatile, greater prediction errors led to a greater phasic pupil response (*t* = 10.01, β = 0.3, SE = 0.003, and *P* < 0.001). This indicates that their importance was up-weighted. Conversely, when the environment was more stable, greater prediction errors led to a reduction in phasic pupil size (*t* = −16.20, β = −0.05, SE = 0.003, and *P* < 0.001). This indicates that their importance was down-weighted. Hence, infants optimized their learning by flexibly weighting the impact of the prediction errors, depending on the volatility of the current environment.

### Individual differences in volatility estimation relate to infant temperament

**Fig. 4.**
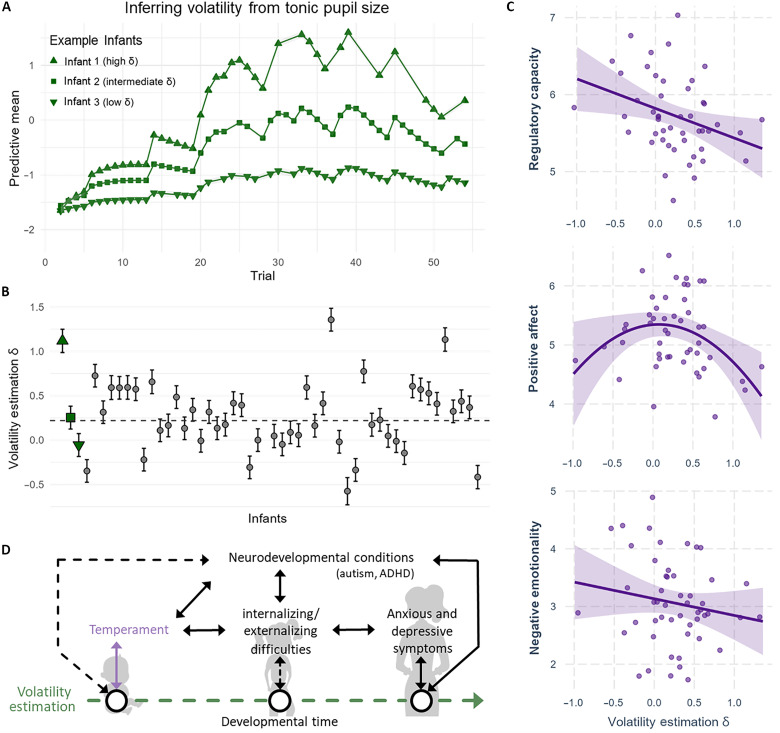
Individual differences in volatility estimation. (**A**) Three example infants displaying volatility overestimation, correct estimation, and underestimation as indexed by high, intermediate, and low δ values, respectively. (**B**) δ mean values and confidence intervals for all infants. The δ values of the three example infants are highlighted in green. The dashed line indicates the group average value of δ . (**C**) Individual differences in volatility estimation were found to be related to regulatory capacity and positive affect, but not negative emotionality. (**D**) Schematic representation of the potential developmental pathway from early volatility estimation abilities to psychological outcomes. Solid arrows indicate associations that were found in previous research, and dashed arrows indicate possible associations; purple arrow indicates the current findings. All arrows are bidirectional as causality cannot be established. Volatility estimation early in life is related to temperament, and it might relate to internalizing/externalizing difficulties in childhood, as well as anxious and depressive traits in adulthood (*35*–*38*). Hence, volatility estimation might offer a transdiagnostic marker that captures the existing longitudinal relations between infants’ temperament, children’s internalizing and externalizing difficulties, and anxious and depressive symptoms and/or neurodevelopmental conditions such as autism and ADHD (*7*, *39*–*43*). This hypothesis awaits empirical testing through longitudinal research.

We tested whether infants differed in their ability to estimate environmental volatility, as such differences might affect how they adapt and react to stimuli and stressors in their daily life. To measure individual differences in volatility estimation, we repeated the analysis relating tonic pupil size to environmental volatility while introducing an individual-level parameter δ for each infant. This parameter captured how strongly each infant’s tonic pupil size correlated with environmental volatility. Hence, higher δ values indicate an overestimation of volatility, while lower values indicate an underestimation of volatility compared to the group average (*32*) (Fig. 4A). Given that infants learn optimally in this task, deviations from average performance correspond to suboptimal performance.

Next, we related these δ values with parental reports of infant temperament, which were obtained through the Infant Behaviour Questionnaire (IBQ). In so doing, we tested whether individual differences in volatility estimation were related to how infants deal with every-day stimuli and stressors early in life. Specifically, we used the three subscales of the IBQ: surgency/positive affect, which reflects the infant’s tendency to engage actively and energetically with their environment; negative affectivity, which indicates how frequently and intensely an infant expresses negative emotions; and orienting/regulatory capacity, which encompasses traits related to attentional control and the ability to soothe oneself. Given that both lower and higher than average scores in volatility estimation might be related to temperament issues, we ran regression models including linear and quadratic terms. We found that volatility estimation as indexed by δ values showed a linear negative correlation with regulatory capacity (*t* = −2.75, β = −1.61, SE = 0.58, and *P* = 0.008), a quadratic relation with positive affect (*t* = −2.43, β = −0.17, SE = 0.07, and *P* = 0.02), and no relation with negative affectivity (*t* = −1.19, β = −0.12, SE = 0.11, and *P* = 0.24). This indicated that infants who overestimate volatility are less well able to regulate their emotions and that infants who are better at estimating volatility show more positive affect.

## DISCUSSION

While previous work has addressed the impact of unpredictable environments on cognitive development, the active role that infants play in responding to environmental volatility was unexplored. Here, we tested infants on a learning task in which volatility levels where systematically manipulated, and we quantified these trial-by-trial changes in volatility with computational modeling. We show that infants have the ability to estimate and adapt to environmental volatility, enhancing their learning and the accuracy of their predictions, but also exhibit substantial interindividual differences in volatility estimation. This variability critically relates to their self-regulation capacities and how they engage with daily stimuli and stressors.

A key strength of this study is the modeling approach, which combined an optimal learner (the VKF) with generalized additive models, relying on a rigorous parameter recovery procedure (see fig. S1). While optimal learner models are typically fitted to behavioral data (e.g., decision responses), here we fitted them to the time course of pupil data instead. This not only allowed us to fit the model to data from infants (who cannot yet produce reliable behavioral responses) but also improved the precision of our estimates due to the higher signal-to-noise ratio of pupil measurements compared to gaze data.

A potential limitation of our design is that we varied the target stimulus on every trial, as the novelty of the stimuli may have introduced additional variability in pupil size. However, this approach allowed us to maintain infants’ attention and collect more than 40 trials per participant on average (as opposed to the typical 5 to 15 trials in habituation studies). These changes in novelty could explain why we observed a slight increase in pupil size, and consequently, in the VKF’s volatility estimates, even during the initial (low-volatility) phase of the task. Crucially, however, our manipulation of volatility is not correlated with time while novelty is, indicating that stimulus novelty alone cannot account for the observed effects on volatility estimation.

When examining infants’ pupil dilation in response to the target appearance, we observed the expected interaction between volatility and prediction error: Larger errors produced greater pupil dilation only under high volatility, consistent with adult studies showing that prior beliefs are updated more rapidly when the environment is perceived as unstable. Under low-volatility conditions, it was low prediction errors, rather than large ones, that elicited stronger pupil responses. One possible explanation is that in stable environments, infants particularly value confirmatory evidence, thereby increasing their physiological arousal when cues confirm existing expectations. Future studies should investigate if this is a fundamental characteristic of infant learning or an idiosyncrasy of our task.

Overall, our results indicate that infants are not only sensitive to changes in environmental volatility but also actively use this information to optimize their learning. As such, these findings promote an outlook on cognitive development where infants and their adaptive skills play an active role in shaping the effects of the environment on their developing minds. In support of this, we found that individual differences in volatility estimation were related to infant temperament, with infants who overestimate volatility showing poorer regulatory capacity and infants who correctly estimate volatility showing more positive affect. In adults, difficulties in adapting to change have been linked to multiple conditions, to the extent that misestimation of volatility has been proposed as a transdiagnostic process that broadly affects mental health (*3*). However, causality remained unclear, as mental health issues could be the underlying cause of volatility misestimation, rather than the result. The relationships we find between infant temperament and volatility estimation points to the possibility that early divergence in learning abilities might be a precursor of symptoms like anxiety and depression. However, a final empirical confirmation of this hypothesis would require longitudinal research.

Revealing how infants estimate and adapt to volatility offers critical insights into the early mechanisms that shape vulnerability and resilience (*17*). Given the active role played by infants in adapting to change, healthy development might not only depend on what kinds of environments infants are exposed to, but also on the infants’ own ability to adapt to them. From a simple change in routines to more fundamental changes in caregivers or social dynamics, it is the infant’s ability to adapt to change that will ultimately shape their experience. Infants who overestimate volatility might struggle even in stable environments, while infants who underestimate volatility might be less able to tackle volatile situations. Conversely, infants who are good at correctly estimating volatility might be better able to flexibly adjust to changing conditions. Hence, it is likely to be the combination of early experiences and the infants’ adaptive learning abilities that together might shape later psychosocial well-being. This hypothesis awaits further verification.

## MATERIALS AND METHODS

### Participants

Infants (*n* = 61, age = 7.7 months, SD = 0.3, and *F* = 29) were recruited via a database of volunteer families. Infants who were born prematurely or had visual impairments were excluded from the recruitment. During the task, trials were presented until the infant lost attention or became fussy. For six infants, data were absent due to fussiness or lack of calibration. In addition, infants with 80% or more missing trials were excluded (*n* = 17) resulting in a final sample of 38 infants. Caretakers of participating infants received either 10 euros or a children’s book as compensation. The study was approved by the faculty’s board of ethics. All caregivers were fully informed about the study procedures and signed an informed consent form before their infants participated.

To replicate the results, we collected an additional sample of infants. Effect sizes indicated that a sample *n* = 20 was necessary. We recruited 30 additional infants (age = 7.7 months, SD = 0.3, and *F* = 12). For two infants, data were absent due to fussiness or lack of calibration. In addition, infants with 80% or more missing trials were excluded (*n* = 8) resulting in a final sample of 20 infants.

The analysis of individual differences in volatility estimation was carried out on both samples combined (*n* = 58). Five infants did not have enough data on both high- and low-volatility trials for the model to converge and were thus excluded from the analysis. For one additional infant, parents did not return the IBQ questionnaire. This resulted in a final sample size of 52 infants (age = 7.6 months and *F* = 23).

### Procedure

Caretakers were asked not to distract or redirect the infant’s attention during the task. Infants were tested in a quiet room without daylight. The infant was positioned in front of the screen, either directly on the caretaker’s lap or in a baby seat at a distance between 60 and 65 cm from the eye tracker. After a five-point calibration, the reversal learning task started. During the task, gaze and pupil data were collected using a Tobii X300 eye tracker with Python via the tobii_research module.

### Materials

Each trial consisted of a fixation bullseye, two cue boxes, and a target, which were presented on a screen at 1920 × 1080 pixel resolution. All images were 250 × 250 pixels and were presented on a gray background (#656565). The fixation bullseye was presented in the center of the screen and the cues and target were presented 300 pixels to the left or the right of the center. The target stimuli consisted of 40 different fantasy figures. The cues were created by scrambling the target stimuli and reshaping them into a square. This way, the luminance of the target and the cue were kept constant.

### Experimental paradigm

The task was programmed in Python 3.6 using PsychoPy software (*33*). As depicted in Fig. 1A, all trials started with the fixation bullseye (3000 ms ± 1000 ms). Then, the cues were presented on the left and right side at the same distance from the center of the screen (2000 ms ± 325 ms). At the start of the cue presentation, a sound was played to signal to the infants the start of a new trial. During the cue presentation, the cues rotated with an angle of 20° and an accompanying sound was played to attract the infants’ attention. Afterward, a target stimulus appeared in place of one of the two cues for 750 ms. The target was also accompanied by a sound. After the target presentation, the same cues were presented statically for 2250 ms.

Four different sequences were generated and they were presented to participants in a pseudo-randomized order. An example of a sequence of trials can be seen in Fig. 1C. The target appeared in a high-likelihood location approximately 90% of the times and in a low-likelihood location the remaining 10% of the times. The high-likelihood location (e.g., left) remained stable for the initial 18 trials. Then, it changed for nine trials (e.g., right) and again for nine more trials (e.g., left). Afterward, the high-likelihood location returned to stable for 18 trials (e.g., right). This manipulation allowed us to vary the trial-by-trial level of volatility as outlined in Fig. 1C. The cycle of stable and changing blocks repeated for as long as the infant remained attentive. If the infant looked away for more than a minute or became fussy, the experiment ended. On average, infants watched 46.12 trials (SD = 19.30), with 71% valid trials (SD = 25%).

### Measures

From the pupillometry data, we extracted a measure of tonic pupil size during the fixation period (i.e., before the trial started) and a measure of phasic pupil size during the target presentation. Phasic pupil size was baseline corrected using the 500 ms preceding the target stimulus presentation. In research with adults, tonic pupil size has been shown to reflect subjective uncertainty (*23*), while phasic pupil size tracks the amount of information contained in a stimulus (*34*) and whether such information is used to improve future predictions (*25*).

From the gaze data, we extracted the proportion of anticipatory looking to each cue location before the target appeared. This measure is widely used as an index of infants’ expectations (*31*) and it was used here to assess whether infants correctly predicted the most likely target location. Specifically, the proportion of anticipatory looking indexed the looking time spent over the left cue, divided by the overall looking time for both left and right cue. We expect this value to be high when infants are anticipating that the target is on the left and low when they expect it to be on the right.

### Computational modeling

A binomial VKF (*22*) was used to track trial-by-trial changes in volatility (Fig. 1B). The model learns the most likely target location via a trial-by-trial updating rule

$$p_{t}=p_{t-1}+\alpha_{t}\left(o_{t}-s\left(p_{t-1}\right)\right)$$

where the probability of where the target will appear (i.e., left or right side) is updated depending on the previous belief $p_{t-1}$ , adjusted by the prediction error (i.e., the difference between the actual outcome $o_{t}$ and the previous belief). The previous belief is mapped to the unit range (i.e., [0,1]) via a sigmoid function *s*. The prediction error is weighted by the learning rate $\alpha_{t}$ , which is computed as follows

$$\alpha_{t}=\sqrt{w_{t}+v_{t}}$$

where $w_{t}$ is the variance of the probability $p_{t}$ , which can be seen as first-order uncertainty (i.e., uncertainty in the outcome), while $v_{t}$ is the volatility of the environment, which can be seen as second-order uncertainty (i.e., uncertainty in whether the environment will change). Volatility is updated on every trial with its own update rule

$$v_{t}=v_{t-1}+\lambda \left(\Delta v_{t}\right)$$

where λ is the learning rate that determines how much volatility prediction errors $\Delta v_{t}$ change previous expectations about volatility $v_{t-1}$ and was introduced in the model as a free parameter. A value of λ = 0 would indicate that environmental volatility is completely disregarded during learning. Conversely, a value of λ = 1 would indicate that the estimates of environmental volatility are fully based on the current moment in time, disregarding the past. Values between 0 and 1 allow for more balanced learning, slowly integrating new information while still maintaining a degree of stability based on past experience.

The value of $v_{t}$ at the start of the task (i.e., when *t* = 0) was introduced as an additional free parameter $v_{0}$ . The free parameters λ and $v_{0}$ were fitted on the basis of the tonic pupil size during the fixation period, such that the relation between volatility and tonic pupil size was maximized (i.e., the negative log-likelihood of the model was minimized) with a grid-search algorithm. We used the following model

$${\text{Pupil}}_{t}=\beta_{0}+\beta_{1}v_{t}+\beta_{2}t+f\left(\text{time},\text{infant}\right)+f\left(x,y\right)+f\left(z\right)+\varepsilon$$

where tonic pupil size on each trial was predicted by volatility estimates $v_{t}$ , trial number t (which allowed us to control for changes in pupil dilation over trials) as well as noise ε . Additional smoothing functions captured constant fluctuations in time across trials (in milliseconds) and infants and changes in pupil size due to eye movements ( x,y ) and distance from the screen ( z).

Before fitting our model to the experimental data, we validated its parameter recovery performance. We generated synthetic tonic pupil data using a range of known λ and $v_{0}$ values and then applied our fitting procedure to estimate these parameters. The procedure successfully recovered the exact simulated parameter values (see fig. S1). Next, we fitted the real data. The lowest negative log-likelihood (−$5.2\cdot 10^{3}$ ) was found for the combination of parameters’ values λ=0.3 and $v_{0}=0.01$ . This was overwhelmingly lower than the negative log-likelihood of a model with in which the value of λ was set to zero (−$3.9\cdot 10^{3}$).

Last, we expanded the group-level model reported above to capture individual differences. We introduced a random intercept for each infant and a random slope δ for volatility. This random slope captured how strongly each infant’s tonic pupil size was modulated by environmental volatility. In this expanded model, δ was estimated, while the parameters λ and $v_{0}$ were fixed at values of 0.3 and 0.01, respectively.

The resulting δ values were used in linear regression models to predict the three subscales of the IBQ (surgency/positive affect, negative affectivity, and orienting/regulatory capacity). The models contained both a linear and a quadratic term, as we expected both high and low levels of δ to be related to poorer IBQ scores.

### Replication

In the additional sample of infants (n = 20), environmental volatility significantly correlated with infants’ tonic pupil size (*t* = 31.62, β = 0.08, SE = 0.003, and *P* < 0.001); the model predictions about the most likely target location significantly correlated with infants’ anticipatory looking (*t* = 4.74, β = 2.97, SE = 0.63, and *P* < 0.001); the interaction between volatility and the magnitude of the prediction errors significantly modulated phasic pupil size (*t* = 36.11, β = 0.34, SE = 0.009, and *P* < 0.001). All the results were thus replicated in this independent sample.
