# Supplementary material for: Volatility-driven learning in human infants
Source: Sci Adv. 2025 Jun 25;11(26):eadu2014. doi: 10.1126/sciadv.adu2014 (PMC12190001; doi:10.1126/sciadv.adu2014)
Supplement: Supplementary file 1 — Fig. S1 [file sciadv.adu2014_sm.pdf]

Science Advances
AAAS

Supplementary Materials for

Volatility-driven learning in human infants

Francesco Poli *et al.*

Corresponding author: Francesco Poli, francesco.poli@mrc-cbu.cam.ac.uk

Sci. Adv. **11**, eadu2014 (2025)
DOI: 10.1126/sciadv.adu2014

This PDF file includes:

Fig. S1

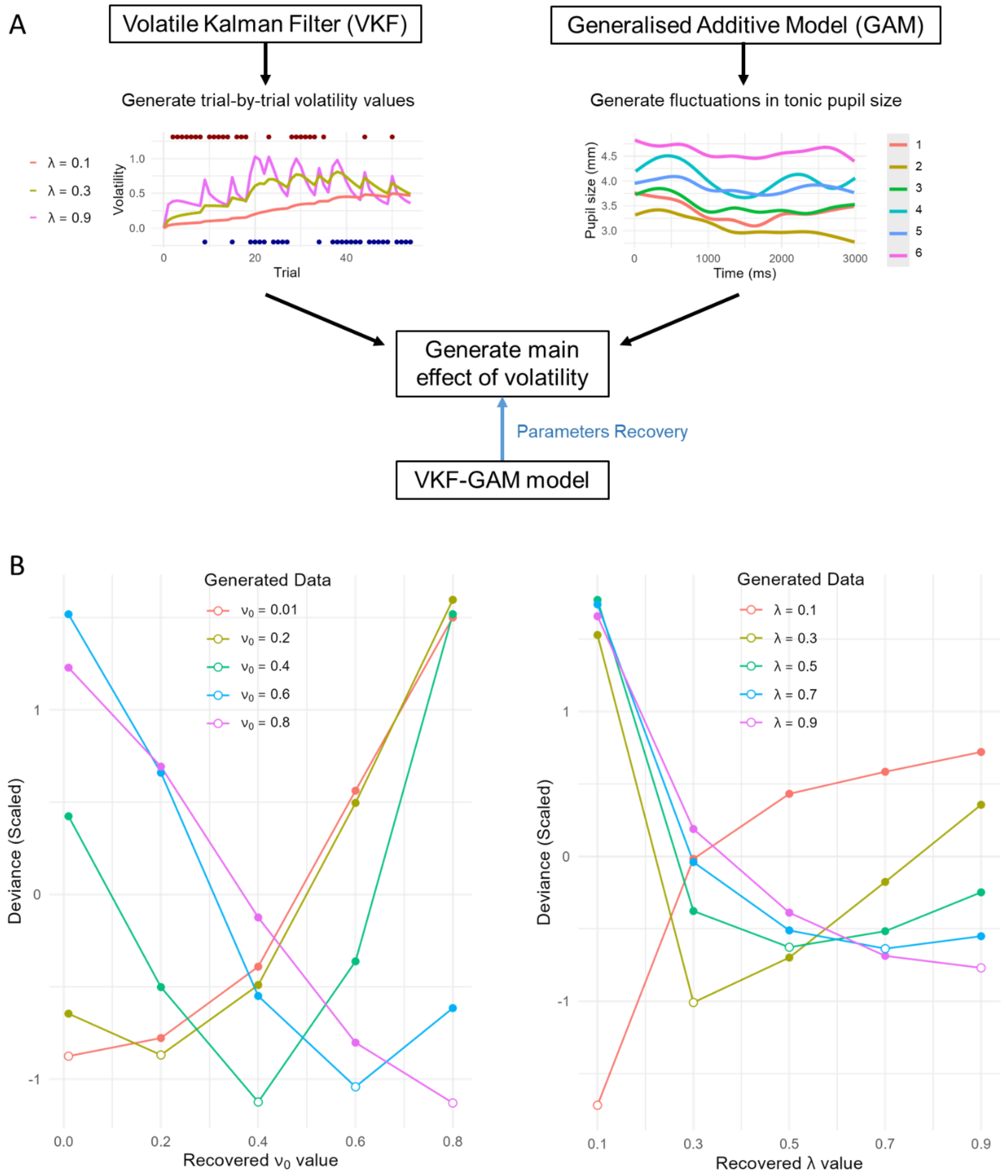

Fig. S1.

Parameter recovery. To assess whether the model parameters λ and v_0 can be reliably estimated from the data, we carried out a parameter-recovery analysis. Panel A displays three representative trial-by-trial volatility trajectories created from distinct combinations of λ and v_0 values. When λ was manipulated, v_0 was fixed at 0.01; when v_0 was manipulated, λ was set to 0.3.

To embed realistic fluctuations in tonic pupil size, we first fit infants' data during the fixation period (≈ 3000 ms) with a generalized additive model that included a linear (parametric) trend over trials in the experiment, a subject-specific smooth change over time within each trial, a two-dimensional smooth adjustment for where the infant was looking on the screen (x-y coordinates), and a smooth adjustment for the viewing distance between eyes and display. The estimated coefficients were then used to generate synthetic pupil data, on which we introduced a linear effect of volatility. These simulated data were analysed with the identical modelling pipeline applied to the empirical dataset in the main article.

Panel B plots the recovered against the generative parameter values. Model deviance (lower = better) served as the goodness-of-fit metric, and values were z-scored within each generative condition. Hollow circles denote, for every generative setting, the parameter value that minimized deviance. For both λ and v_0 there was perfect correspondence between the generative and recovered values. For example, when volatility levels were generated setting $\lambda = 0.3$, the recovered value (i.e., the value that led to best goodness of fit) was 0.3.
